# Reconstruction Error of Calibration Volume’s Coordinates for 3D Swimming Kinematics

**DOI:** 10.2478/v10078-011-0037-6

**Published:** 2011-10-04

**Authors:** Pedro Figueiredo, Leandro Machado, João Paulo Vilas-Boas, Ricardo J. Fernandes

**Affiliations:** 1 Centre of Research, Education, Innovation and Intervention in Sport, Faculty of Sport, University of Porto, Porto, Portugal.; 2 Porto Biomechanics Laboratory, University of Porto, Porto, Portugal

**Keywords:** accuracy, reliability, digitisation, swimming, kinematics

## Abstract

The aim of this study was to investigate the accuracy and reliability of above and underwater 3D reconstruction of three calibration volumes with different control points disposal (#1 - on vertical and horizontal rods; #2 - on vertical and horizontal rods and facets; #3 - on crossed horizontal rods). Each calibration volume (3 × 2 × 3 m) was positioned in a 25 m swimming pool (half above and half below the water surface) and recorded with four underwater and two above water synchronised cameras (50 Hz). Reconstruction accuracy was determined calculating the RMS error of twelve validation points. The standard deviation across all digitisation of the same marker was used for assessing the reliability estimation. Comparison among different number of control points showed that the set of 24 points produced the most accurate results. The volume #2 presented higher accuracy (RMS errors: 5.86 and 3.59 mm for x axis, 3.45 and 3.11 mm for y axis and 4.38 and 4.00 mm for z axis, considering under and above water, respectively) and reliability (SD: underwater cameras ± [0.2; 0.6] mm; above water cameras ± [0.2; 0.3] mm) that may be considered suitable for 3D swimming kinematic analysis. Results revealed that RMS error was greater during underwater analysis, possibly due to refraction.

## Introduction

When analysing human movement, it is a common practice to measure the position of significant body landmarks to determine the movement kinematics (Challis, 1995). This approach has been applied to a wide variety of problems (Chen et al., 1994), particularly to evaluate the above and underwater swimming stroke (Figueiredo et al., 2009).

Analysis of multi-planar activities engage three-dimensional (3D) reconstruction, frequently using the direct linear transformation algorithm by transforming two-dimensional image coordinates – DLT (Chen et al., 1994; Allard et al., 1995; Challis, 1995), as proposed by Abdel-Aziz and Karara (1971). With the DLT technique, an appropriate number of points with known 3D coordinates on a calibration volume are used as control points for the calibration of the recording space. In this procedure, the number and distribution of the control points, as well as the size of calibration volume, affect the reconstruction accuracy (Lam et al., 1992; Chen et al., 1994).

For aquatic propelling purposes, swimmers must constantly interact with water. However, since it is a complex and highly integrated form of movement, all the immersed and emerged body parts play a key role in this sport. The kinematic analysis of the swimming locomotion impose obstacles to data acquisition, particularly by the existence of errors associated to image distortion, digitisation and 3D reconstruction (Payton and Bartlett, 1995; Kwon and Casebolt, 2006); thus, it seems important to observe its influence on the final results, analysing validity, reliability, and accuracy (Scheirman et al., 1998; Hopkins, 2000). When referring to underwater 3D kinematic analysis, regardless of the equipment used (underwater housing, underwater windows or periscope systems), refraction implies higher reconstruction error (Yanai et al., 1996; Lauder et al., 1998; Kwon, 1999; Kwon and Lindley, 2000).

Three-dimensional reconstruction has been frequently used in swimming studies (Cappaert et al., 1995; Payton and Bartelett, 1995; Berger et al., 1999; Figueiredo et al., 2009). However, the study of its accuracy has been scarce (Psycharakis et al., 2005; Gourgoulis et al., 2008). The purpose of this study was to assess the influence of the number of control points in the accuracy of the under and above water 3D reconstruction. In addition, the influence of the control location was also assessed, and both environments were compared.

## Material and methods

**Figure 1:**
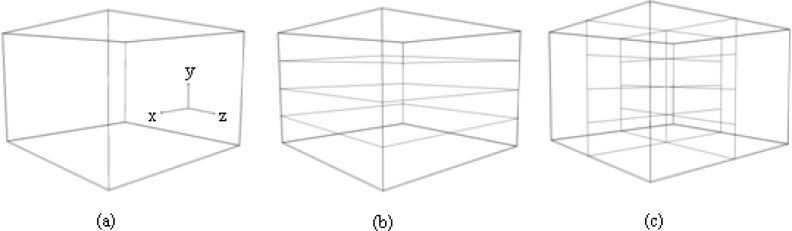
Calibration volumes: (a) calibration volume #1 where the control points are distributed on vertical and horizontal rods; (b) calibration volume #2 where the control points are distributed on vertical and horizontal rods and facets; (c) calibration volume #3 where the control points are distributed on crossed horizontal rods.

Recordings of the different control points distribution in a calibration volume (Figure 1) were carried out simultaneously by four under and two above water cameras (Sony® DCRHC42E). The volumes were positioned half above and half below water surface, in a 25 m swimming pool. The cameras were mounted at an equal distance from the centre of the calibration volume, and their optical axes formed an angle of ∼100° between the axes of the two above water cameras; the angle between below water cameras varied from ∼75° to 110°. A LED system visible in the field of view of each camera was used for its temporal synchronisation. Cameras were placed at 1.0 to 1.5 m depths to avoid errors due to its axes being in the same reference planes of the volume. The above water cameras were placed at height of 3.0 to 3.5 m.

All calibration volumes were made-up from 1 cm diameter aluminium tubing, being 3 × 2 × 3 m in the horizontal (x), vertical (y) and lateral (z) directions, respectively. The size of the calibration frame was established to allow a complete stroke cycle of front crawl swimming.

To assess the number of control points required to maximise the accuracy of 3D coordinate reconstruction, 12 markers in the calibrated space were digitised over 50 frames for each underwater and above water camera views. Seven series of digitising were performed for this set of 12 markers, using 8, 12, 16, 20, 24, 28 and 30 control points, both for above and below water. In addition, the used validation points did not serve as control since the DLT algorithm is optimised for its reconstruction (Challis and Kerwin, 1992; Chen et al., 1994; Kwon, 1999).

All reconstruction errors were calculated from the raw coordinate data without any smoothing procedure (Scheirman et al., 1998), and determined by the Root Mean Square (RMS) error of the 12 validation points (for each calibration volume) using the following equations:

(1) $$Ex_{r}=\sqrt{\frac{\sum_{t=1}^{N}{\left(x_{nt}-x_{t}\right)}^{2}}{N}}$$

(2) $$Ey_{r}=\sqrt{\frac{\sum_{t=1}^{N}{\left(y_{nt}-y_{t}\right)}^{2}}{N}}$$

(3) $$Ez_{r}=\sqrt{\frac{\sum_{t=1}^{N}{\left(z_{nt}-z_{t}\right)}^{2}}{N}}$$

(4) $$E_{r}=\sqrt{\frac{\sum_{t=1}^{N}{\left(x_{nt}-x_{t}\right)}^{2}+{\left(y_{nt}-y\right)}^{2}+{\left(z_{nt}-z_{t}\right)}^{2}}{N}}$$

where *E_Xr_*, *E_Yr_*, *E_Zr_* and *E_r_* were the RMS errors for each axis and for the resultant error, respectively; *x_ni_*, *y_ni_* and *z_ni_* were the real coordinates; *x_i_*, *y_i_* and *z_i_* were the reconstructed coordinates; and *N* was the number of points used. To obtain reliability estimation, one operator (to avoid any inter-operator errors) repeated the procedure 10 times, being considered as the standard deviation value across all digitisation of same the marker.

## Results

**Figure 2:**
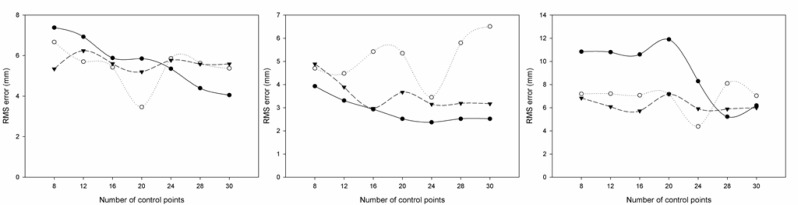
Underwater RMS errors for the x (left panel), y (centre panel) and z (right panel) axes for the different calibration volumes (#1 - solid line, #2 - dotted line and #3 - dashed line)

Figure 2 presents the RMS errors for the x (left panel), y (centre panel) and z (right panel) coordinates, for different numbers of underwater control points in the three studied calibration volumes.

**Figure 3:**
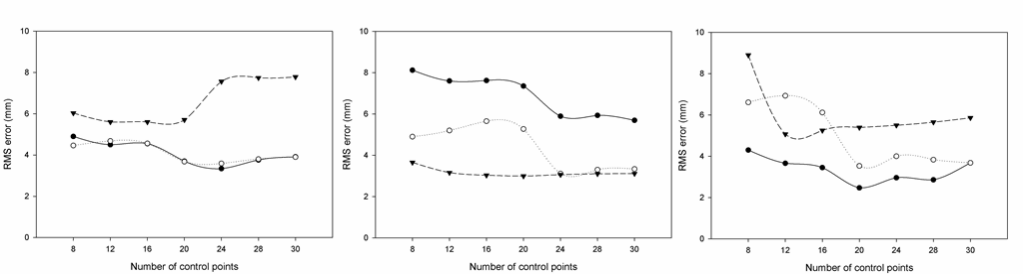
Above water RMS errors for the x (left panel), y (centre panel) and z (right panel) axes for the different calibration volumes (#1 - solid line, #2 - dotted line and #3 - dashed line)

Figure 3 shows the RMS errors for the x (left panel), y (centre panel) and z (right panel) coordinates, for different numbers of control points above water in the three studied calibration volumes.

The resultant RMS errors are presented in Table 1, being possible to observe higher underwater values comparing to the above water values.

The reliabilities of one marker varied between ± [0.2; 0.6] mm for the underwater cameras, and between ± [0.2; 0.3] mm for the above water cameras.

## Discussion

The results of the present study revealed that for the underwater recordings accuracy increased as the number of control points augmented (until 20–24, depending of the studied volume), as reported before (Lauder et al., 1998; Psycharakis et al., 2005). Regarding the above water recordings, accuracy also increased with the number of the control points (8 to 20–24), as reported by Chen et al. (1994) and Shapiro (1978). A further increase until 30 points did not improve the accuracy of both measurements.

The calibration volume #2 showed lower resultant RMS error for under and above water environments, representing 0.2 % of the calibrated space for each underwater axes, and 0.1, 0.2 and 0.1 % of the calibrated space for the x, y and z above water axes.

Considering the volume of the calibrated space, the errors were similar or lower than those reported previously. For the underwater environment Payton and Bartlett (1995) reported values of 2.3, 3.3 and 2.9 mm, while Lauder et al. (1996) observed RMS values ranging from 1.86 to 2.82 mm (lateral axis), from 4.53 to 7.32 mm (horizontal axis) and from 3.51 to 7.76 mm (vertical axis). Psycharakis et al. (2005) presented RMS error values of 3.9, 3.8 and 4.8 mm for the x, y and z axes respectively, representing 0.1, 0.2 and 0.5 % of the calibrated space. Payton et al. (2002) reported mean errors of 1.5 to 3.1 mm for a 1.1 m^3^ volume (representing 0.2 % of the calibrated space for each direction). Kwon et al. (1995), for a calibration volume of 3 × 1 × 1 m, referred RMS values of 6.4, 6.6, 4.2 mm for x, y and z axes, respectively. Gourgoulis et al. (2008), presented for a small (1 × 1 × 1 m) and large (1 × 3 × 1 m) calibration volume, RMS values of 1.61 and 2.35 mm (lateral axis), 2.99 and 4.64 mm (horizontal axis) and 2.83 and 2.59 mm (vertical axis), respectively.

For above water reconstruction, Coleman and Rankin (2005) studied the golf swing and reported RMS errors of 5.1 to 9.8 mm (representing 0.4, 0.5 and 0.3 % of the calibrated space, for the x, y and z axes, respectively). Challis (1995) presented values ranging from 6.1 to 23.0 mm (calibration volume with 1 × 1 × 0.6 m of dimensions), while Chen et al. (1994), for a calibration volume of 2.10 × 1.35 × 1.00 m, found a mean error ranging from 1.8 to 3.6 mm for x, 1.9 to 2.7 mm for y, 5.4 to 12.8 mm for z, and a resultant from 6.6 to 1.6 mm, depending on the number of control points used. In addition, Yanai et al. (1996) reported mean resultant errors ranging from 8.34 to 16.44 mm for the above and from 9.93 to 16.22 mm for the below water control volumes (1.5 × 8.4 × 2 m).

The higher RMS errors observed in the horizontal axis, independently of the recording environment, are in agreement with the literature (Lauder et al., 1996; Yanai et al., 1996). According to Chen et al. (1994), the greater reconstruction error in the horizontal axis could be attributed to the cameras’ set-up regarding to the calibration volume, i.e. when the angle between the optical axes of the cameras is low, the resolution of a given distance on the image plane is different in the horizontal than in the other two axes. Consequently, random errors during the digitisation cause higher reconstruction errors in the longitudinal axis. However, the angles used in the present study ranged between 75 and 110º, which are higher than those used by Chen et al. (1994) and Gourgoulis et al. (2008): 35 and 41º, respectively. These higher RMS values in x axis occurred only for some of the control points series (Figure 2).

The present results revealed that during underwater recordings the RMS reconstruction errors were greater comparing to those obtained above the water, independently of the calibration volume used, which is in accordance with the literature (Yanai et al., 1996; Lauder et al., 1998; Gourgoulis et al., 2008). These increased reconstruction errors, when underwater recordings were analysed, were probably due to light refraction (Lauder et al., 1996; Lauder et al., 1998; Kwon and Lindley, 2000; Kwon and Casebolt, 2006). Furthermore, according to Kwon and Lindley (2000), during the calibration of the underwater space, the real 3D coordinates of the control points are forced to fit to the deformed image-plane coordinates. Although this mismatch error could be evenly distributed through the control volume, its maximum values (calibration error) normally occur at the boundary of the control volume, due to the non-linear distortion caused by refraction (Kwon and Lindley, 2000). In addition, the observed results pointed out a good reliability, since small errors were found in our study. In fact, the reliability of the coordinate reconstruction was similar (or even better) than the values reported by Psycharakis et al. (2005): ± 0.4, ± 0.5 and ± 0.4 mm, for the x, y and z axes, respectively.

The results of this study indicated that the reconstruction errors were higher in underwater than above water environment. However, in both conditions, the magnitude of the reconstruction errors may be considered suitable for 3D swimming kinematic analysis. Complementarily, in spite of a lower resultant RMS error of the calibration volume #2, the choice of the number of control points and corresponding location should consider the specificity of the aquatic activity; for instance, calibration volume #3 could be used for synchronised swimming since its actions are mostly in y and z axes, in which the volume #3 presented low RMS error values.

## Figures and Tables

**Table 1:** Resultant RMS errors for underwater and above water recordings for the #1, #2 and #3 calibration volumes

Number of control points	Underwater			Above water		

	#1	#2	#3	#1	#2	#3
8	7.38	6.19	5.69	5.17	5.32	6.19
12	7.01	5.80	5.40	5.25	5.60	4.61
16	6.47	5.97	4.76	5.21	5.44	4.62
20	6.76	5.33	5.34	4.51	4.16	4.69
24	5.34	4.56	4.94	4.06	3.57	5.37
28	4.04	6.51	4.88	4.18	3.64	5.49
30	4.25	6.30	4.92	4.43	3.64	5.58
